# Enamel surface roughness following debonding resin clean up using high speed air-turbine and electric handpieces. In vitro study

**DOI:** 10.1186/s12903-024-04339-x

**Published:** 2024-05-25

**Authors:** Tarek N. Yousry, Salma Abolgheit, Hassan E. Kassem

**Affiliations:** 1 Orthodontic Department, Alexandria University, Alexandria, Egypt; 2 Biomaterials Department, Alexandria University, Alexandria, Egypt

**Keywords:** enamel roughness, electric handpiece, air turbine, orthodontic debonding, orthodontic polishing

## Abstract

**Background:**

High speed electric handpieces have recently been growing in popularity among dental professionals. Advantages include smoother surface preparation and increased cutting efficiency.

**Aim:**

The primary objective was to compare enamel surface roughness following resin cleanup after bracket debonding using highspeed air turbine versus electric handpiece. The secondary objective was to record the time needed for resin-clean up.

**Method:**

Forty deidentified freshly extracted human premolars were cleaned and sectioned at the cement-enamel junction. The crowns were embedded in acrylic blocks. Enamel surface roughness parameters (Ra, Rz, Rp and Rv) were measured using a stylus profilometer. Brackets were bonded using a light-cure orthodontic adhesive and stored in distilled water for 24 h. Following bracket debonding, the specimens were randomly divided into 2 groups: First group: resin clean-up was carried out using a 12-fluted carbide bur mounted on a high-speed air turbine; and second group: where an electric handpiece was used. Surface roughness parameters were measured following resin clean up and after polishing using pumice and a rubber cup. Time needed for resin clean-up was recorded. Differences in enamel surface roughness and time between groups were compared using repeated measures ANOVA and independent samples t-test, respectively at *P* ≤ 0.05.

**Results:**

The electric handpiece groups showed significantly higher values for Ra, Rz and Rp both following resin cleanup and polishing. Time taken for resin cleanup was significantly longer for the electric handpiece group.

**Conclusion:**

Considering both surface roughness and time, electric handpiece do not seem to add greater effectiveness or efficiency to resin cleanup following orthodontic bracket debonding.

## Introduction

Orthodontic treatments, particularly when combined with periodontics or restorative procedures, are increasingly popular due to their ability to enhance both aesthetics and long-term dental health through interdisciplinary approaches [1]. One of the principal tenets of any medical practice is to “Do no harm.” The primary objective of adhesive removal following bracket debonding at the conclusion of orthodontic treatment is to return the enamel surface back to its pre-treatment state.

Following the introduction of the acid etch technique and bonded bracket to Orthodontics by Newman [2], there has been a substantial increase in the tools for post-debonding resin clean up and enamel surface polishing. The classical technique involves the use of dome-tapered tungsten carbide bur in a contra-angle handpiece operating at 30,000 rpm for rapid adhesive removal without enamel damage [3]. The introduction of fine fluted carbide bur has allowed the use of high-speed handpieces for resin clean up [4]. A recent survey of the active members of the American Association of Orthodontists have demonstrated that most of the respondents uses 12-fluted carbide bur mounted on high- speed air turbine handpiece followed by polishing with pumice and a rubber cup on a slow-speed air turbine [5]. Tungsten carbide burs operated in either high-speed or slow-speed settings are the most popular rotary instrument used in orthodontic clinics for resin clean-up [6]. 

Recently, there has been an increasing trend in dentistry to shift from air-turbine to high- speed electric handpieces [7]. Despite their popularity in other parts of the world, high speed electric handpieces have struggled to end the US market. The general preference has been for the air-turbine handpiece for most procedures [8]. 

Possible advantages of the high-speed electric handpiece that may be relevant to the orthodontic profession include the low noise, less vibration and high concentricity and more precision. In addition, there is the added benefit of adjusting the number of revolutions per minute (rpm) per the manufacturer’s instructions for the bur used [9]. Greater cutting efficiency and less increase in pulp chamber temperature have been reported when cutting with a high-speed electric handpiece irrespective of the rotary instrument whether carbide bur or diamond point [7, 10]. Electric handpieces were shown to produce smoother tooth surface preparation compared to air-turbine handpieces regardless the grit of the rotary cutting instrument used tooth preparations for fixed prosthetics [11]. This may prove advantageous in restoring a smooth enamel surface following resin clean up after debonding of orthodontic brackets or in clear aligner therapy attachment removal.

Studies investigating resin cleanup after bracket debonding have used high [5, 12] or slow-speed handpieces [13, 14] with tungsten carbide burs. However, there is usually no mention of whether the handpiece was operated on an air turbine or an electric motor [15]. 

Hence, the primary objective of the study was to test the null hypothesis that there is no statistically significant difference in enamel surface roughness after post debonding resin clean up and polishing when resin cleanup is carried out by high-speed air turbine or electric contra-angle high speed handpiece. The second null hypothesis tested was that there is no difference in resin cleanup time between the two instruments.

## Materials and methods

Forty freshly extracted maxillary premolars were used in this study. The teeth were collected from patients treated in the orthodontic department, Faculty of Dentistry, Alexandria University whose premolars were to be extracted for orthodontic purposes. The research protocol was approved by the Research Ethics Committee of Alexandria University Faculty of Dentistry (IRB No. 001056 – IORG 0008839) prior to any research-related activities. All research activities involving human specimens followed with the Declaration of Helsinki [16] and the domestic laws [17]. All specimens were deidentified and no potentially identifying patient information was collected in relation to the specimens.

The sample size estimation was conducted using G* power software version 3.1.9.6, (Universität Kiel, Germany), using a between-factor repeated measure ANOVA at α = 0.05 and power = 0.8 [18]. An average of Ra for 2 groups over 3 time points were calculated from Eliades et al. [19] yielding an effect size of f = 0.39 yielding a total sample size of 38 specimens with an actual power of 81.7%. The sample size was increased to 40 to allow for outliers or possible non-reads by the profilometer.

A standard experimentation protocol for enamel surface roughness measurement after resin clean-up was followed in this study [19]. The teeth were scaled, polished with pumice and a rubber cup. Roots were sectioned, pulp remnants removed from the crown and subsequently disinfected in 70% ethanol.

The sectioned crowns were embedded in acrylic resin cylinders of 20 mm diameter x 10 mm height. The cylinders were coded for identification purposes. To standardize the area of surface analysis, four shallow indents were made with a carbide bur corresponding to the corners of an imaginary square centered in the middle third of the buccal surface of the crown.

The designated region of interest was subjected to profilometric analysis using a stylus profilometer (Mahrsurf PS10, Mahr, Germany) with the contact stylus perpendicular to the specimen surface. At each timepoint, two profilometric readings were recorded and the average of the two readings for each parameter was calculated.

The following roughness parameters were measured:

1. Ra – The average surface roughness which is defined as the arithmetic mean of absolutes distances from the center line within the measuring length.
2. Rz - Average maximum peak to valley of five consecutive sampling lengths within the measuring length.
3. Rp – Maximum profile peak height.
4. Rv – Maximum profile valley depth.

Following the baseline profilometric analysis, enamel surface was etched with 37% phosphoric acid for 30 s, rinsed and dried with a two-way air water syringe. Modified edgewise metal brackets (Modern Orthodontics, Ludhiana, India) were bonded using a light cure orthodontic adhesive resin (Transbond XT, 3 M Unitek, Manrovia, CA, USA) according to the manufacturer’s instructions. The specimens were stored in sterile water at 37° for one week prior to debonding. Brackets were debonded by a debonding plier by the same operator.

The specimens were randomly divided into two groups. The random sequence was generated in Microsoft Excel (Microsoft corporation, Washington, USA) [20]. The adhesive remnant was removed using a friction-grip12-fluted carbide bur Adhesive remover H22AGK, Komet dental, Brasseler GmbH, Lemgo, Germany). In the first group, an air-turbine high-speed handpiece (T3 Racer, Sirona, Bencheim, Germany) was used operating at 2.2 bar pressure to deliver 160,000 rpm. In the second group, an electric high-speed “red” contra-angle handpiece (XM-L0105, Westcode, Foshan Guangdong, China) on an electric motor (NL 400-1, Westcode, Foshan Guangdong, China) was used set at 120,000 rpm as per manufacturer’s instructions for both handpieces for the adhesive remover bur. A new bur was used for every 10 teeth. The resin cleanup was performed under the operatory light until no visible resin remnant was visible to the naked eye. The time needed for complete resin removal was determined by an investigator other than the operator blinded to the allocation of the specimens using a stopwatch.

The specimens were subsequently polished with a rubber cup and pumice using a slow speed handpiece (NSK, Nakanichi Inc, Tochigi, Japan) operating on an air-turbine at 1.5 bar pressure to deliver 3000–5000 rpm.

In addition to the baseline measurement, surface roughness parameters were recorded following resin-clean up and after polishing. All roughness parameters were measured by one investigator blinded to the allocation of the specimens.

### Statistical analysis

The normality assumption was tested using histograms and Shapiro Wilk test. Parameters of surface roughness were compared between the groups using repeated measures ANOVA adjusted for baseline values followed by Bonferroni post hoc tests using Statistical Package for Social Sciences SPSS Version 21.0 (IBM Corp, Armonk, NY, USA). The time needed for resin cleanup was compared using independent samples t test. The level of significance was set at *P* ≤ 0.05.

## Results

**Fig. 1:**
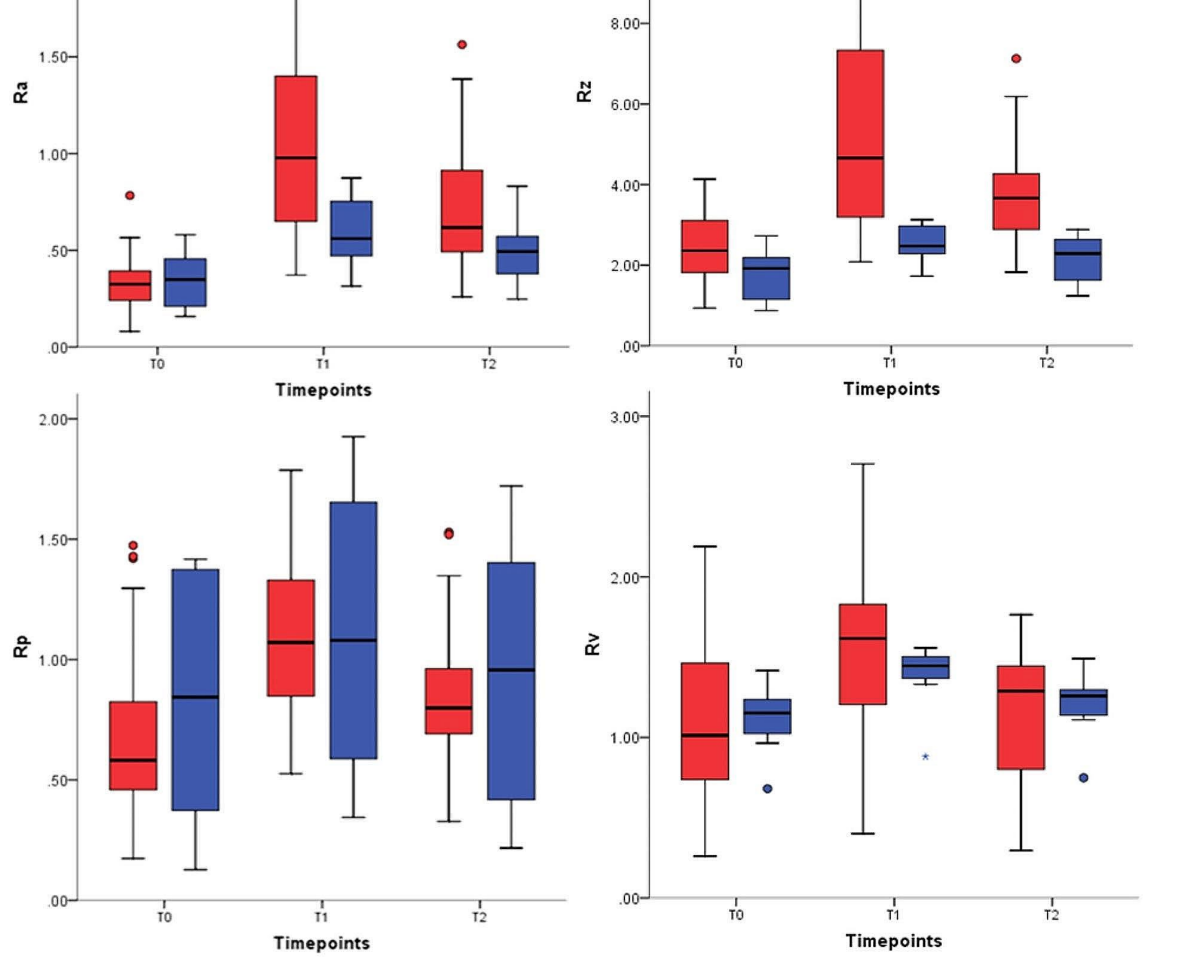
Box and plot charts of the surface roughness parameters Ra, Rz, Rp, and Rv in Electric handpiece and Air turbine groups at T0, after prophylaxis; T1, after resin cleanup; and T2, after polishing

**Fig. 2:**
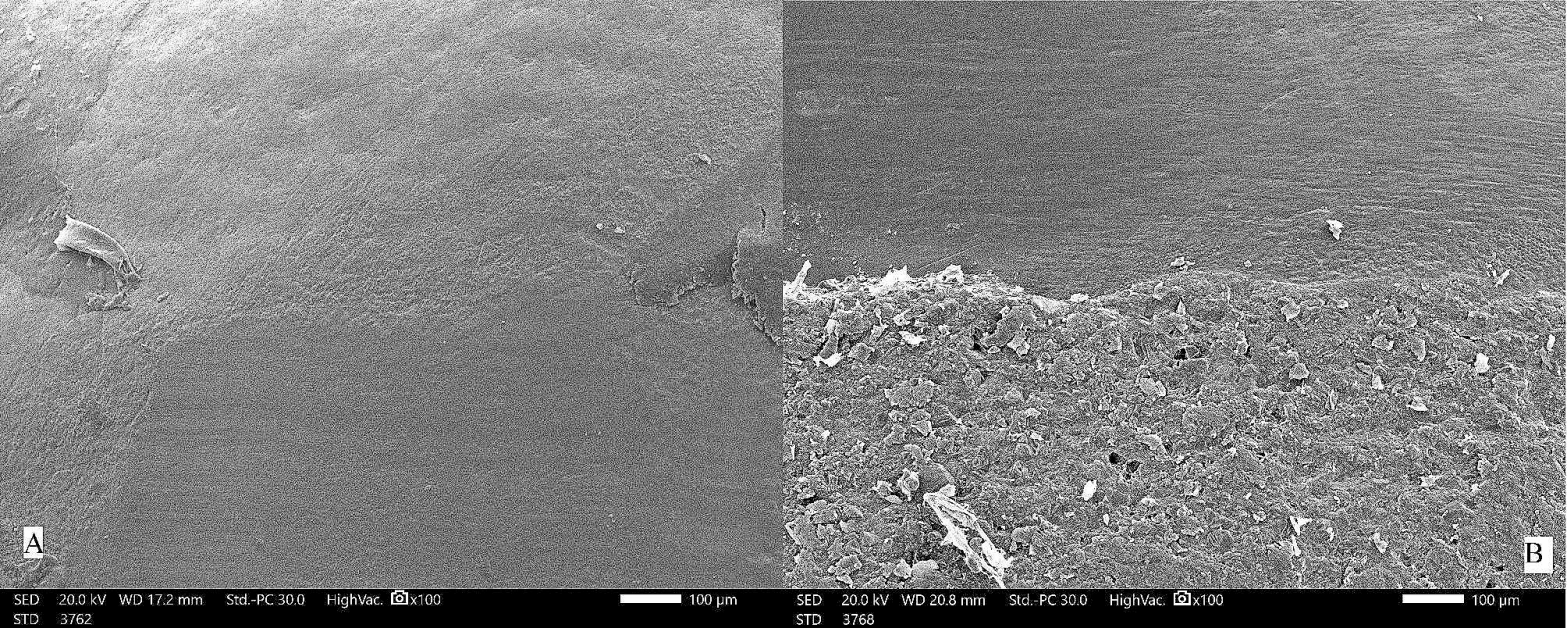
Representative SEM pictures of A, electric handpiece; and B, Air turbine following resin cleanup and polishing. There is no clear distinction between the polished surface and the surrounding enamel in A. In B, the polished surface is distinctly difference in topography with a distinct line of demarcation with the surrounding enamel. (Magnification = x 100)

Mean and standard deviations of roughness parameters are shown in Table 1. All parameters followed the same pattern of increase following resin-cleanup and decrease following polishing yet not approximating baseline values. Figure 1. The electric handpiece showed statistically significant greater values in all roughness parameters except for Rv both following resin-cleanup and polishing. The greatest mean difference was observed in Rz both following resin-cleanup (3.33, C.I. [2.24,4.41]), and polishing (1.54, C.I. = [1.01,2.07]) Table 2. Time for resin-cleanup was significantly longer in the electric handpiece group with a mean difference of 3.72 s (C.I. [1.24,6.20]) Table 3. Representative scanning electric microscope of specimens from both groups following polishing are shown in Fig. 2.

**Table 1:** Descriptive statistics of surface roughness parameters (Ra, Rz, Rp, Rv) in Electric handpiece (Electric) and Air turbine (Air) groups

	Time points						
		T0		T1		T2	
		Mean	S.D.	Mean	S.D.	Mean	S.D.
Ra	Electric *n* = 20	0.29	0.15	1.23	0.42	0.82	0.29
	Air *n* = 20	0.36	0.13	0.62	0.25	0.51	0.20
Rz	Electric *n* = 20	2.62	0.99	6.37	2.09	4.30	1.09
	Air *n* = 20	1.97	0.66	2.77	0.80	2.37	0.64
Rp	Electric *n* = 20	0.62	0.32	1.07	0.33	0.80	0.27
	Air *n* = 20	0.84	0.44	1.14	0.45	0.96	0.45
Rv	Electric *n* = 20	0.91	0.49	1.49	0.71	0.97	0.38
	Air *n* = 20	1.28	0.25	1.55	0.22	1.36	0.23

Ra, Rz, Rp, Rv, Surface roughness parameters.

T0, Before bonding; T1, after resin; T2, after polishing.

**Table 2:** Comparison of surface roughness parameters (Ra, Rz, Rp, Rv) after resin cleanup (T1) and after polishing (T2) between the electric handpiece (Electric) and air turbine (Air) groups

	Electric – Air (*n* = 20)					
	Time points					
	T1			T2		
	Mean Difference	95% C.I.	*P*†	Mean Difference	95% C.I.	*P*†
Ra	0.61	[0.38,0.85]	0.015*	0.33	[0.16,0.50]	0.001*
Rz	3.33	[2.24,4.41]	0.001*	1.54	[1.01,2.07]	0.001*
Rp	0.17	[0.02,0.31]	0.023*	0.96	[0.01,0.18]	0.037*
Rv	0.17	[-0.20, 0.55]	0.354	0.24	[-0.12, 0.07]	0.625

Ra, Rz, Rp, Rv; Surface roughness parameters.

T1, after resin cleanup; T2, after polishing.

† Based on repeated measures ANOVA adjusted for T0. Post hoc comparison using Bonferroni correction.

* Statistically significant at *P* < 0.05.

**Table 3:** Comparison of time for resin cleanup between the Electric handpiece (Electric) and Air turbine (Air) groups

Time for resin cleanup (seconds)					
	Mean	S.D.	Mean Difference	95% C.I.	*P†*
Electric *n* = 20	19.27	4.50			
Air *n* = 20	15.54	3.07	3.72	[1.24,6.20]	0.004*

† Based on independent samples t test, equal variances not assumed

* Statistically significant at *P* < 0.05

## Discussion

The clinical search for an efficient and safe protocol for clean-up of residual orthodontic resins from the enamel surface resulted in the development of various methods, such as: hand instruments, burs (carbide, diamond and composite), discs, rubbers, stones, ultrasonic tools, lasers, and air abrasion techniques [21, 22]. Regardless of the technique used, still most instruments used to remove resin residues after debonding may scratch the enamel due to their shape and sharpness [23]. 

Despite its increased popularity, this study may well be the first to investigate the use of electric handpiece in removal of orthodontic adhesives remnants following debonding. This increased shift towards use of electric handpieces is attributed to its constant torque which eliminates the stalling or reduced speeds experienced when using an air-driven handpiece to cut through teeth, crowns or other dense materials. The constant torque produces a concentric cutting motion as speed is maintained [24]. 

The null hypotheses tested in the present study were that no statistically significant differences existed between the high speed air turbine and the electric handpiece in enamel surface roughness following resin clean up after bracket debonding or the time needed for resin cleanup. Both null hypotheses were rejected. Most enamel surface roughness parameters were significantly greater in the electric handpiece compared to the air turbine. Time needed for resin cleanup was significantly shorter with the air-turbine handpiece.

The profilometric analysis results showed that the air turbine showed smoother surface than the electrical handpiece. Also, the results of profilometric analysis for the air turbine handpiece showed an even smoother surface than the baseline measurements (sound enamel). The air turbines are lighter and offer a greater sense of grip and flexibility [25]. This might offer better control for experienced orthodontists during finishing providing smoother surface. This might in part describe the smoother surface produced by the air turbine handpieces.

Another important point to be considered is the torque of the micromotors, which is the force that generates rotation of the device. It directly influences the load, which is the quantitatively measured force applied to the tooth or any surface being worked on [26]. The load produced by the electric micromotor is greater than that of the pneumatic equipment, varying between 3% [27] and 2.32% [10]. Although Christensen [8] suggested that the constant torque and lack of ‘stalling’ of the electric handpiece make it more efficient at cutting than the air-turbine, its use for finishing rather than cutting might offer different results. Since orthodontic bonding composite are considerably softer than dental structure [28], the use of the electric handpiece for residual resin removal following orthodontic brackets debonding is expected to be significantly lower load than its use in tooth preparation. The lower load required may explain the varying result of this study compared to the use of electric handpiece in crown preparation.

There are conflicting reports in the literature regarding the effect of vibrations from dental handpieces. It has been suggested that such vibrations may be responsible for symptoms of hand-arm vibration in dentists and may cause enamel cracking in teeth [29]. Controversially, Watson et al. [30] reported no statistical difference in the amount of enamel cracking between the air-turbine and electric handpiece. Similarly, the surface roughness of the prepared teeth and the crown fit between the tooth and ceramic crown were not affected by the air-turbine or electric handpiece [25]. 

Regarding the time needed for resin cleanup, the air turbine group showed significantly shorter time needed to clean all residual adhesive compared to the electric handpiece. This result seems intuitive considering the higher speed recommended by the manufacturer operating the resin cleanup bur on air turbine. Bonding composite being a softer substrate compared to the tooth enamel may also obviate the greater efficiency of the electric handpiece reported in tooth preparation studies [31]. However, saving approximately 2 min in resin cleanup of both arches may not be the weighting factor favoring one finishing method over other.

A possible limitation of this study is the lack of volumetric analysis of the enamel surface to quantify enamel loss during resin cleanup and polishing. In addition, better measures could have been taken to ensure complete resin cleanup and preservation of sound enamel such as using color changing bonding composite or performing the procedures under loop magnification. Future research may investigate the effect of using different rpms in the performance of the electric handpiece and whether the experience of the operator with the electric handpiece may have an effect on the time needed for resin cleanup.

## Conclusion

Considering both surface roughness and time, the electric handpiece does not seem to add greater effectiveness or efficiency to resin cleanup following orthodontic bracket debonding compared to the air-turbine handpiece.

## Data Availability

The raw data is availabe on request .
